# Alteration in ACL loading after total and partial medial meniscectomy

**DOI:** 10.1186/s12891-024-07201-x

**Published:** 2024-01-25

**Authors:** S. Uzuner, L. P. Li

**Affiliations:** 1 Department of Mechatronics, Faculty of Engineering, University of Duzce, Konuralp Campus, 81620 Duzce, Marmara Türkiye; 2 Department of Mechanical and Manufacturing Engineering, University of Calgary, 2500 University Drive, N.W, Calgary, AB T2N 1N4 Canada

**Keywords:** ACL mechanics, Creep loading, Finite element, Knee laxity, Poromechanical model

## Abstract

Anterior cruciate ligament (ACL) injuries are often caused by high impact loadings during competitive sports but may also happen during regular daily activities due to tissue degeneration or altered mechanics after a previous knee injury or surgery such as meniscectomy. Most existing research on ACL injury has focused on impact loading scenarios or the consequence of ACL injury on meniscus. The objective of the present study was to investigate the effects of varying degrees of medial meniscectomy on the mechanics of *intact* ACL by performing a poromechanical finite element analysis under moderate creep loadings. Four clinical scenarios with 25%, 50%, 75% and total medial meniscectomy were compared with the intact knee finite element model. Our results suggested that different medial meniscal resections may increase, at different extents, the knee laxity and peak tensile stress in the ACL, potentially leading to collagen fiber fatigue tearing and altered mechanobiology under normal joint loadings. Interestingly, the ACL stress actually increased during early knee creep (~ 3 min) before it reached an equilibrium. In addition, meniscectomy accelerated ACL stress reduction during knee creep, transferred more loading to tibial cartilage, increased contact pressure, and shifted the contact center posteriorly. This study may contribute to a better understanding of the interaction of meniscectomy and ACL integrity during daily loadings.

## Introduction

ACL ruptures are common knee injuries in young, physically active individuals. Approximately 100,000 to 200,000 ACL injuries are encountered annually in the United States [1]. ACL injuries are seen at high incidence in sports that involve pivoting and accelerating or decelerating, such as football, basketball, and team handball. The ligament is frequently subjected to dynamic loads during these sports activities [2]. Although ACL injuries are primarily seen in activities of player-to-player contact (42.8%), they also may occur without contact (37.9%) [3]. On the other hand, ACL injury is a common pathological issue that may be associated with meniscectomy. In fact, ACL deficiency was observed in half the meniscectomized patients [4]. While the ACL is the tissue primarily responsible for the anteroposterior (AP) and rotatory laxity of the knee joint [5], the menisci, whose main task is to resist compression forces, also contributes significantly to knee stability [6]. Several in-vivo [7, 8] and in-vitro [9–11] studies have revealed the significance of the meniscus in limiting the anterior tibial translation (ATT). ATT has been reported to increase by 3 to 7 mm after meniscal resection on ACL-injured knee joints [7, 12–15]. In addition, it was emphasized that repairing torn meniscus with suturing remarkably reduces ATT as compared to meniscectomy [16]. Consequently, ACL injuries may not simply occur at rapid and high loads during intensive physical activities. An inclusive perspective might be that ACL deficiency may also occur in impaired knees, e.g., due to meniscectomy, even when high loading is absent [7, 17–19]. Moreover, 77% of patients presenting with acute traumatic hemarthrosis of the knee induced by causes such as meniscal tears are believed to have an ACL injury, even without readily apparent joint laxity [20–24].

Most published finite element studies tried to understand the effect of ACL deficiency on knee joint mechanics using single-phase elastic models that neglected fluid flow [25–27]. This model simplification is likely valid when examining the effect of instantaneous loads that may occur in extreme exercise activities on the ACL [15]. However, using a single-phase model to simulate daily activities ignores fluid pressurization and load redistribution in the joint, which can be phenomenal within seconds of a load application. In fact, fluid pressure and flow could have a crucial role in the knee joint’s biomechanical behavior, given the significant poromechanical behavior exhibited by cartilaginous tissues [28]. Moreover, the fluid flow in the soft tissue induced by loading causes stress redistribution and facilitates nutrient transport in the knee joint. Therefore, it may add knowledge to ACL injuries under various scenarios, if the creep behavior is determined to show the effect of moderate loads on ACL mechanics. These forces are smaller than impact forces experienced in competitive sports, but more frequently experienced by the ACL and thus may have a greater impact on its mechanobiology.

In brief, the mechanism between previous meniscectomy and subsequent ACL deficiency remains to be further clarified, because published studies have focused only on the consequence of ACL deficiency to subsequent meniscal injury or concurrent ACL and meniscal injuries [15, 29]. Furthermore, the current literature lacks information on the effect of load redistribution in the knee joint on ACL mechanics, associated with fluid pressurization in the intact and meniscectomized knees. In an attempt to bridge this knowledge gap in ACL mechanics, the objective of the present study was to discover the effect of various medial meniscectomies on the alteration of loading in healthy ACL during the creep of moderate knee loadings. Our study may provide insight into the cause of ACL fatigue during low loading activities, which is commonly associated with meniscectomy. We hope to contribute to the growing understanding of the pathologic factors that can lead to ACL tears.

## Methods

### Intact and meniscectomized knees

**Fig. 1:**
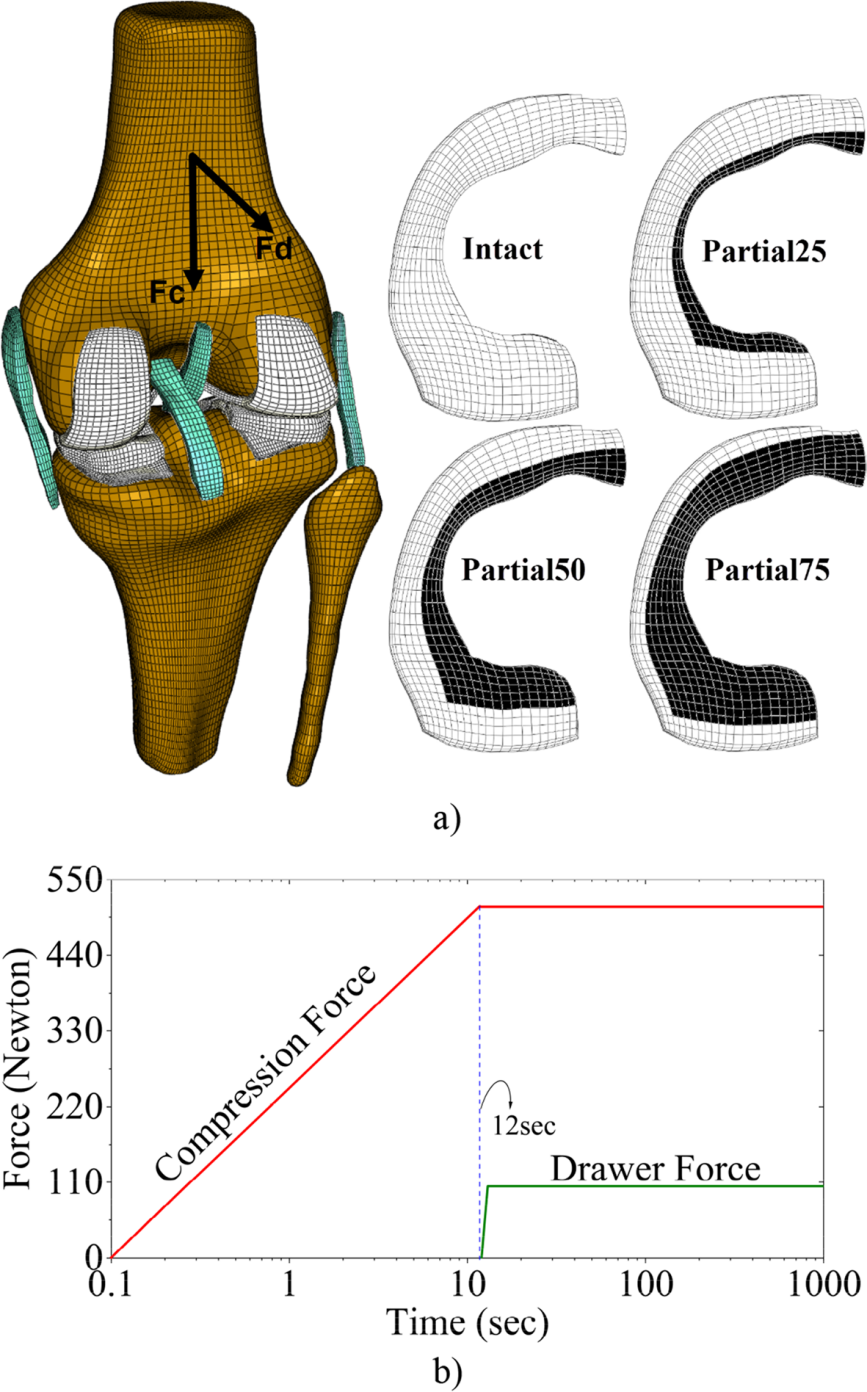
Schematic of finite element model of the knee and loading: **a** knee, intact medial meniscus and three cases of partially resected medial meniscus; **b** ramp and creep loading protocol. The black area indicates the amount of medial meniscectomy with approximately 25, 50 or 75% resection in the three cases. The total medial meniscectomy was also modeled. F _c_ and F _d_ denote the compressive and drawer forces, respectively. The ramp compression force reached the maximum at 12 s, while drawer force was applied at 12 s and ramped in 1 s

The 3D finite element (FE) *intact* knee model was previously reconstructed with 3T MRI of the right knee from a 25-year-old male participant weighing 68 kg with no history of leg deformities [30]. The tibiofemoral joint model consisted of distal femur, proximal tibia and fibula, all cartilaginous tissues in the lateral and medial compartments, and the two cruciate and two collateral ligaments (Fig. 1a). Articular cartilage and menisci were modeled with four and five layers of elements, respectively. All tissues were discretized using hexahedral elements (Fig. 1a), which normally deliver better accuracy for pore pressures than tetrahedral meshes. Furthermore, hexahedral elements facilitate faster convergence in contact modeling. Pure hexahedral porous elements with 20 nodes (C3D20RP in ABAQUS) were used for the tibial cartilage, whereas 8-node elements were used for the menisci and femoral cartilage (C3D8RP). Although C3D20RP elements show better pressure distribution than C3D8RP, they require more computational time. Solid element type C3D8 was used for ligaments. Because ligaments are subject to tensile forces, fluid pressure can be neglected. Rigid element type R3D4 was used for bones because the bony structures are much stiffer than soft tissues. The performance of mesh was evaluated in a previous study [31]. Similar meshes were evaluated and found to yield good numerical results in the previous study [30]. Furthermore, the intact model was validated with 10-minute in-vivo measurement of the same participant using high-speed video-radiography.

It is believed that medial meniscectomy has a greater effect on altered joint mechanics than lateral meniscectomy [9, 29, 32–34] which is attributed to the firmer attachment of the medial meniscus to the tibial plateau by capsular components [35]. On the other hand, the lateral meniscus may bear a greater portion of the vertical force shared by the lateral compartment than the medial meniscus does in the medial compartment [36, 37]. The present study addressed compromised knee joint mechanics due to medial meniscectomy because of its more frequent prevalence as compared to lateral meniscectomy and the function of medial meniscus as a secondary restraint to ATT [38]. Thus, only the effect of medial meniscectomies on ACL mechanics was investigated in this study, including four clinical scenarios similar to that evaluated by a team of 12 orthopedic surgeons and researchers [39]. The sizes and locations for partial meniscectomy are shown in Fig. 1a, which was implemented by removing the corresponding elements (dark in Fig. 1a) that were carefully meshed to represent 25, 50, and 75% of the meniscus as similarly defined in the reference [39]. The mechanical changes in the ACL were evaluated by comparing the intact and meniscectomized knees.

### Soft tissue model

The solid phase was modeled by introducing fibrillar and non-fibrillar matrices to consider their distinct properties: the collagen fibrillar matrix is critical in resisting tensile loadings, while the proteoglycan matrix plays an essential role in compressive forces. The fibril-reinforced poromechanical model was previously published [40] and provided here briefly for easy reading. The equation below defines the tensile stress (Cauchy stress) that arises in the fibril matrix when subjected to a load,

1 $${{\upsigma }}_{x}^{\textrm{f}}\left(\text{t}\right)={{\upsigma }}_{x}^{\text{f}}\left(0\right)+{\int }_{0}^{\text{t}}{\textrm{G}}_{x}\left(\text{t}-{\uptau }\right)\frac{\partial {{\upsigma }}_{x}^{\text{e}}}{\partial {{\upepsilon }}_{x}} \frac{\partial {{\upepsilon }}_{x}}{\partial {\uptau }}\text{d}{\uptau }$$

which is a function of time associated with the reduced relaxation function denoted with ${\text{G}}_{x}$. Collagen fiber orientation was implemented based on the split-line pattern [41]. Equation (1) was defined concerning the local *x* axis, but a similar formulation was applied in the *y* and *z* directions. The elastic response in Eq. (1) was defined as

2 $${{\upsigma}}_{\text{x}}^{\text{e}} \left({\upepsilon}\right)= {\text{A}}_{\text{x}}{{\upepsilon }}_{\text{x}}+{\text{B}}_{\text{x}}{{\upepsilon }}_{\text{x}}^{2}$$

Here, ${\text{A}}_{\text{x}}$ and ${\text{B}}_{\text{x}}$ are material constants acquired from the uniaxial tensile tests [42, 43]. The reduced relaxation function took the following form,

3 $${\text{G}}_{x}\left(\textrm{t}\right)=1+\sum _{\text{m}}{\textrm{g}}_{\text{x}}^{\textrm{m}}\text{exp}\left(^{-\text{t}}\! / \!_{{{\uplambda }}_{\text{x}}^{\textrm{m}}}\right)$$

where ${\text{g}}_{\text{x}}^{\text{m}}$ and ${{\uplambda }}_{\text{x}}^{\text{m}}$ represent, respectively, the weight constants and characteristic times for the viscoelastic dissipation. The Neo-Hookean hyperelastic model was used to describe non-fibrillar matrices.

4 $${{\upsigma}}^{\text{n}\text{f}}= \frac{2{\text{C}}_{10}}{\text{J}}\left(\overline{\mathbf{B}}-\frac{1}{3}\text{t}\text{r}\left(\overline{\mathbf{B}}\right)\mathbf{I}\right)+\frac{2}{{\text{D}}_{1}}\text{J}\left(\text{J}-1\right)\mathbf{I}$$

${\text{C}}_{10}$ and ${\text{D}}_{1}$ are material constants [42, 43]. In Eq. (4), $\mathbf{I}$ is the unit tensor, J stands for Jacobian, and $\overline{\mathbf{B}}$ is the distortional component of the left Cauchy-Green deformation tensor [35]. Darcy’s law is used to describe the solid-fluid interaction in soft tissues,

5 $${{\varnothing}}^{f}{v}_{x}={-k}_{x}{p}_{x}$$

Here $k$ and $p$ are permeability and fluid pressure, respectively. The void ratio, ${\phi ^f}$, is assumed to be the ratio of fluid and solid volumes.

Ligaments were modeled with the same material law, neglecting fluid pressurization. The material properties of the soft tissues used in the present study were derived from the in-vivo measurement performed on the same participant in the previous study [30] and provided in Table 1. When fitting the in-vivo measurement to extract the properties, past in-vitro test data from the literature were referenced in order to choose the range of variation appropriately. The tissue model has been validated with various experiments and the material properties shown in Table 1 were found to be reasonable [44, 45].

**Table 1:** Material properties of fibrillar and non-fibrillar matrices in all soft tissues

	Fibrillar matrix Eqs. (1,2)				Non-fibrillar matrix Eq. (4)	
	Primary fibrillar direction (*x*) (MPa)		Perpendicular directions (*y*,*z*) [MPa]		C_10_ (MPa)	D_1_ (MPa^-1^)
	A	B	A	B		
Femoral Cartilage	2	1000	0.9	480	0.096	0.310
Tibial Cartilage	2	1000	2	1000	0.096	0.310
Menisci	12	1500	2	750	0.183	0.595
Ligaments	46	1118	0	0	0.769	1.660
Weight constants (g^m^), characteristic times (λ^m^)	Eq. (3)				g^1^=0.870; λ^1^=10 g^2^=0.036; λ^2^=100 g^3^=0.273; λ^3^=1000	
Permeability [mm^4^/Ns]	Eq. (5)				k_*x*_= 0.002, k_*y*_=k_*z*_=0.001	

### Loading and boundary conditions

Body weight induces both vertical compression and drawer forces within the knee [46]. The compression force of 520 N and drawer force of 104 N were applied to the femur at full extension (Fig. 1b). The compressive force was the ground reaction force obtained during the participant’s 10-minute standing in an in-vivo experiment [30]. The drawer force was calculated as 20% of the compression force, which was suggested in a published study [47]. While the drawer force was applied to the anterior-posterior, the compression force was involved in the proximal-distal directions. In the present study, to maintain unconstrained mobility by avoiding any adverse consequences of load positioning on joint kinematics, the femur was set free to translate all three directions while not allowed to rotate in any direction. No rigid-body motion was allowed in the tibia and fibula. These boundary conditions may be sufficient to understand the fundamental function of ACL in restricting ATT [5]. The TIE contact definition option in ABAQUS was used to attach the femoral cartilage to the femur, the tibial cartilage to the tibia, the meniscal horns to the tibial plateau, and lastly, the ligaments to the corresponding bones. Six contact pairs were defined among the meniscus, femoral, and tibial cartilages. Three were in the lateral, while the others were in the medial compartment. Contact pairs were defined to simulate the mechanical interactions among the menisci, femoral, and tibial cartilages. A surface-to-surface hard contact was used with the linear penalty method. The contact pairs were specified with a friction coefficient of 0.02, as established in the literature [48]. The large deformation option, NLGEOM in ABAQUS, was utilized to integrate geometric nonlinearity into the finite element analysis. Pore pressure was set to zero as the initial condition to allow fluid to exit from the non-contact surface of soft tissues.

The pre-strain was 2% for medial and lateral collateral, 2.5% for anterior cruciate, and zero for posterior cruciate ligaments, as pre-strains in ligaments may alter the load distribution in the knee joint [49]. The knee model was validated with the in-vivo study on the same man’s knee [30].

## Results

**Fig. 2:**
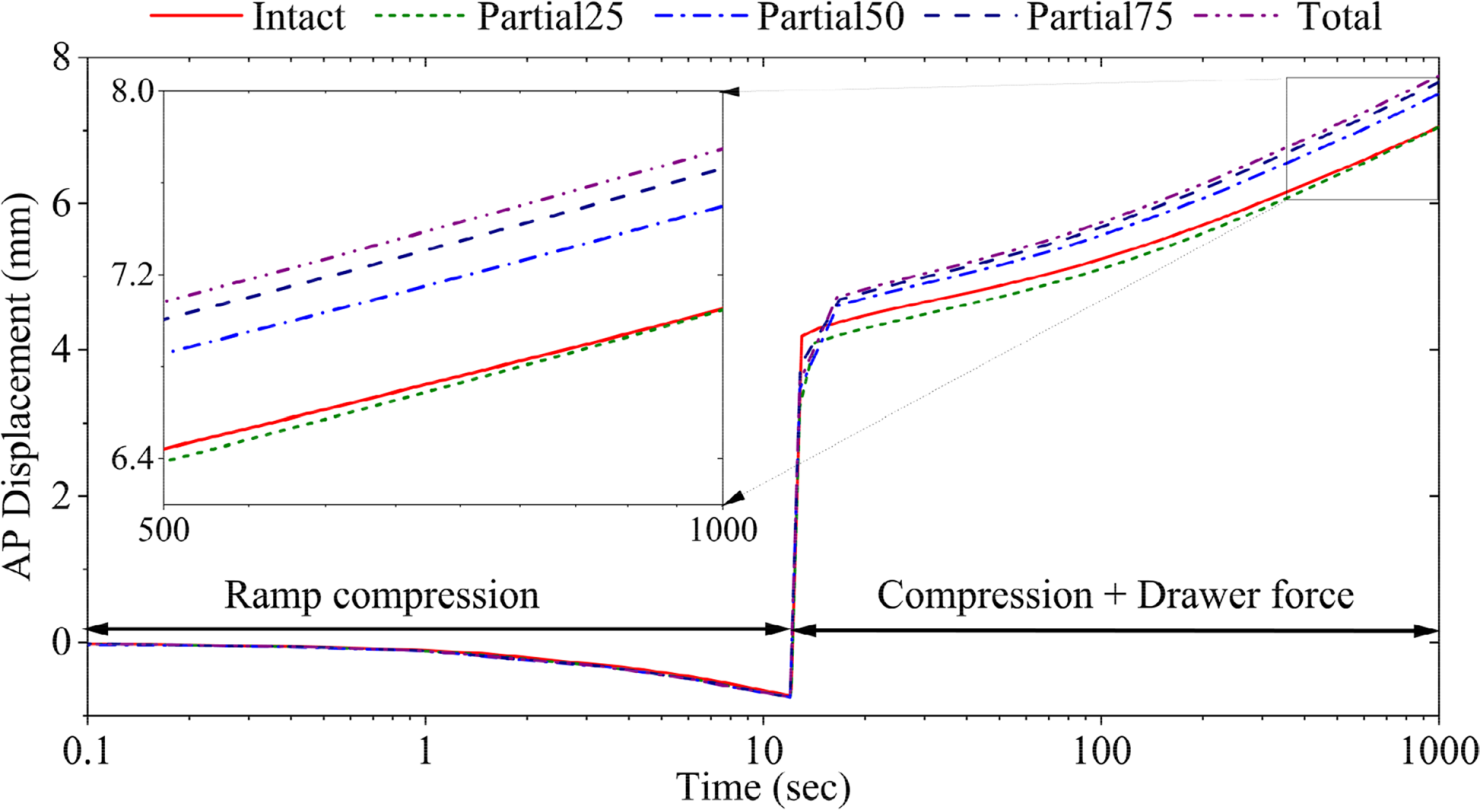
Anterior-posterior femoral translation of intact and medial meniscectomized knees under the combined forces. On the vertical axis, the negative values depict the anterior direction, while the positive values represent the posterior direction

With the compression force alone, the femur in all knee models moved anteriorly (Fig. 2), noting that the tibia was fixed in the modeling. However, the femur in all knee models started to move posteriorly with the addition of the drawer force, and continued to move posteriorly during creep till equilibrium. The increase in resection of the medial meniscus did not change the anterior femoral translation during the compression force but increased posterior femoral translation with the addition of the drawer force. Posterior femoral translation increased by 6.2% in partial50, 8.5% in partial75, and 9.8% in total meniscectomized knees at the 1000th second (Fig. 2).

**Fig. 3:**
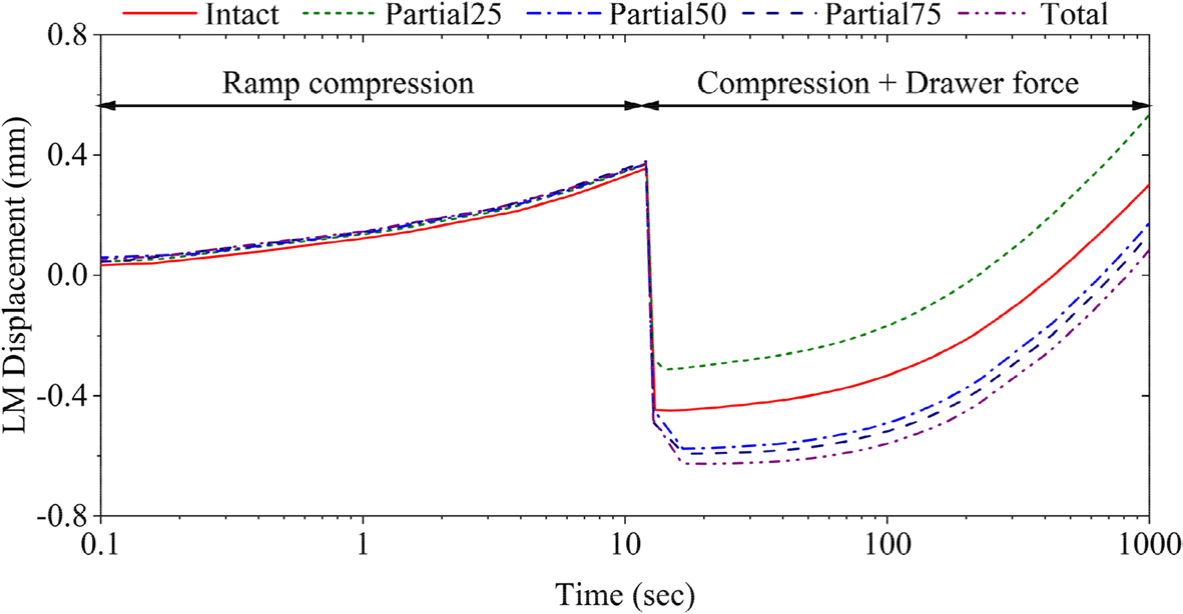
Lateral-medial femoral translation of intact and medial meniscectomized knees under the combined forces. On the vertical axis, the negative values depict the lateral direction, while the positive values represent the medial direction. The displacement was increased above 1.5 mm for all cases at 10,000 s when the equilibrium was far from reached

During the ramp compression (0-12 s), the femur shifted towards the medial side in all knee models (Fig. 3). Total medial meniscectomy caused the femur to move 4.6% more to the medial side than the intact case at the end of ramp compression. However, the application of the drawer force caused the femur in all models to slide to the lateral side. Then, the femur in all models shifted to the medial side again as creep developed. Increasing the amount of meniscal resection increased the displacement of the femur towards the lateral at 60 s by 39% in partial50, 46% in partial75, and 55% in total meniscectomized knees (Fig. 3).

**Fig. 4:**
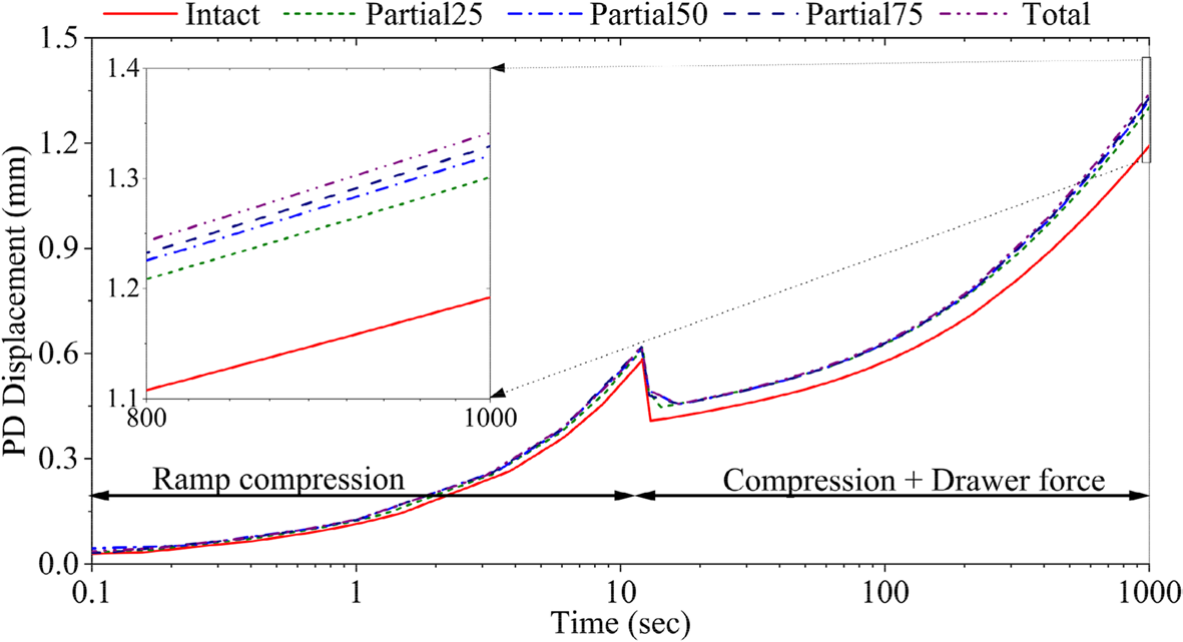
Proximal-distal femoral translation of intact and medial meniscectomized knees under the combined forces. On the vertical axis, the positive values represent the downward direction

The increase in the medial meniscal resection increased the vertical displacement of the femur (Fig. 4). At 12 s, the vertical displacement of the femur increased by 5.5% in partial25, 6.5% in partial50, 7.1% in partial75 and 7.6% in total meniscectomized knees, as compared to the intact knee. The drawer force augmented these differences. The distal movement (downward) of the femur at 1000 s was increased by 9.2% in partial25, 11.3% in partial50, 12.2% in partial75, and 13.0% in total meniscectomized knees, as compared to the intact knee joint.

**Fig. 5:**
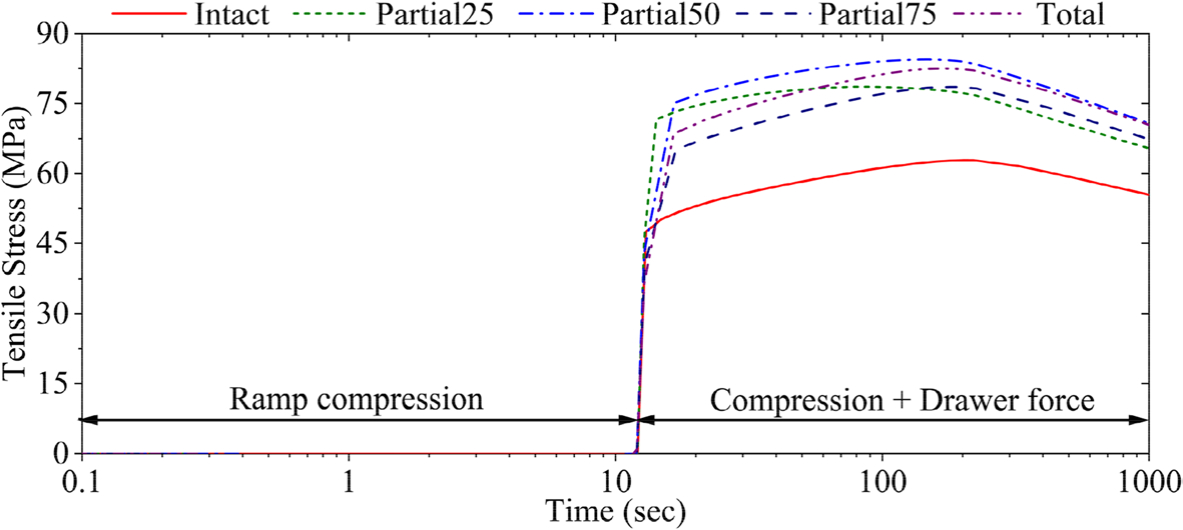
Tensile stress of ACL in the longitudinal direction. The peak tensile stress for each model may occur at different times

**Fig. 6:**
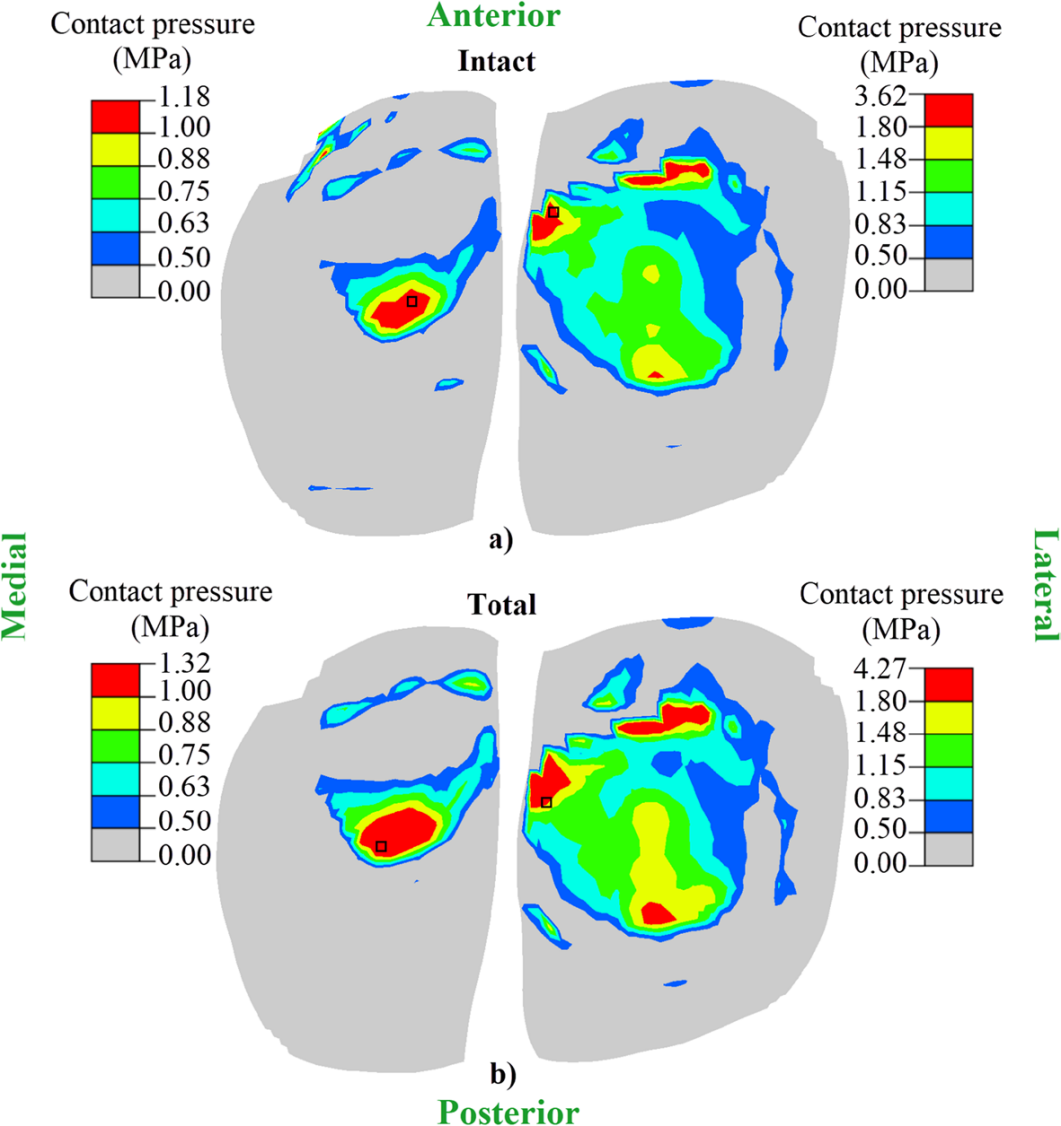
Contact pressure distribution in tibial cartilage in (**a**) the intact and (**b**) total meniscectomized knees at 60 s. The black square shows the location of peak contact pressure. Superior view

The compressive force naturally did not produce much tensile stress in the ACL (Fig. 5). The tensile force in the ACL started to increase with the application of drawer force. As the amount of medial meniscal resection increased, the stress in the ACL increased. Compared to the case of the intact knee, the peak ACL tensile stress increased by 25% in partial25, 34.5% in partial50, 25% in partial75, and 31.5% in total medial meniscectomized knees. The standard deviation in ACL tensile stress of the partial meniscectomy models compared to the intact model was 9.03, while that of the total meniscectomy was 13.77. This means that the effect of total meniscectomy was more significant than that of partial meniscectomy. The peak tensile stresses occurred at different times in each case. Total medial meniscectomy elevated the maximum contact pressures in both tibial plateaus and further augmented the difference between the two compartments (Fig. 6b vs. a).

## Discussion

While literature studies have focused on cartilage deformation after ACL injury, the present study has examined the effect of knee laxity resulting from altered cartilage contact on the mechanics of *intact* ACL, which were predicted with FE simulations of meniscectomy at different degrees. The unique outcomes of the present study may be summarized as follows.

1. Knee laxity increased as the amount of meniscectomy increased even when the ACL remained intact (Figs. 2, 3 and 4). The peak tensile stress in the ACL increased accordingly with the amount of meniscectomy (Fig. 5), which could have implications in collagen ruptures in the ACL under normal joint loadings.
2. The ACL stress increased during the early knee creep regardless of meniscectomy before decreasing toward equilibrium (Fig. 5), which indicated the necessity of creep modeling used in the present study.
3. Meniscectomy accelerated ACL stress reduction during knee creep (Fig. 5), forcing the tibial cartilage to absorb more loading, and thus increasing the maximum contact pressure with a shift in its contact center to the posteromedial side.

Augmenting the meniscal resection resulted in an increased anterior-posterior femoral translation, which was increased by 9.5% at 20 s in the total medial meniscectomized knee as compared to the intact knee (Fig. 2). Similarly, an increased resection of the medial meniscus led to more lateral-medial translation of the femur (Fig. 3). For example, at 60 s, the femur shifted 55% more laterally in the total meniscectomized knee comparing to the intact knee (Fig. 3). The differences in the translations predicted for different models became smaller with creep, e.g., the difference between the intact and partial50 models was 28.9% at 16 s, whereas 6.6% at 1000 s. The increased meniscal resection also increased the vertical displacement of the femur (Fig. 4). At 1000 s, the vertical displacement in the total meniscectomized model increased by 12.5% compared to that of the intact model. In conclusion, knee stability was compromised more with the amount of meniscal resection.

The translation of the femur was reversed in all three directions once the drawer force was added (ramped from 12 to 13 s) (Figs. 2, 3 and 4), which may not have been captured with an elastic analysis. In particular, the femur moved anteriorly with the compressive force only but posteriorly consistent with the direction of the drawer force (*t* ≥ 12 s) (Fig. 2). The motion of the femur in the other two directions was only reversed momentarily during the ramp loading phase of the drawer force (12–13 s) and resumed the original moving directions during creep (*t* ≥ 13 s) (Figs. 3 and 4), another indication of necessary creep analysis.

The overall ACL strain was around 5.5% (Table 2) in all knee models and did not differ significantly with meniscectomy, but there was a substantial difference in the local strain at the ACL insertion point to the femur (36% in intact vs. 43% in total). The differences in the local stress were shown in Fig. 5. This result may help us understand why 77% of patients with acute traumatic knee hemarthrosis may have experienced ACL injuries [20–23], even though it is not evident during clinical assessment [24]. The larger medial meniscal resection, the more increased ACL stress, e.g., total medial meniscectomy increased ACL tensile stress by 30% (Fig. 5). These results may explain meniscectomy-related ACL tears.

**Table 2:** Comparison of the predicted anterior tibial translation and ACL strain at 13 s, when the drawer force reached the maximum, with the in-vivo and in-vitro experimental results for the intact knee joint. APFT = anterior-posterior femoral translation

	Method	AP force (AP moment)	Compression force	APFT	ACL strain (%)
Present study	FEA	108 N (45Nm)	520 N	4.2 mm	5.5
Papageorgiou et al. [50]	Experimental	134 N	200 N	4.9 mm	―
Lin et al. [51]	Experimental	134 N	490 N	6.0 mm	―
Fleming et al. [52]	Experimental	(24Nm)	240 N	―	3.1
Asaeda et al. [53]	Experimental	146 N	732 N	4.2 mm	―
Rudy et al. [54]	Experimental	100 N	nil	5.4 mm	―

A reduction in peak tensile stress of 13.5% occurred in the ACL of the intact joint during knee creep from 180 to 1000 s (Fig. 5). In comparison, it was around 18% in meniscectomized knees (Fig. 5). Accelerated ACL stress reduction during the creep of the meniscectomized knees may mean a larger load redistribution in cartilaginous tissues as indicated by the contact pressure changes (Fig. 6). Peak contact pressure increased by 12.9% in the medial and 18% in the lateral cartilages with total medial meniscectomy as compared to that of the intact knee.

The maximum contact pressure in the lateral was about three times higher than that in the medial tibial cartilages (Fig. 6 at 60 s), because the drawer force moved the femur toward the medial side (Fig. 3) and thus shifted the contact center of the lateral tibial plateau. The contact center was formed at the point where the lateral tibial cartilage contacted the lateral intercondylar eminence (Fig. 6a, b). As the femur moved toward the medial side (Fig. 3), the part of lateral tibial cartilage mating the intercondyloid eminence was subjected to more stress than that in the medial cartilage. Moreover, since medial meniscectomy increased the displacement of the femur to the posteromedial side (Figs. 2 and 3), it also shifted the contact pressure to the posteromedial side (Fig. 6a vs. b).

The knee joint is a complex structure, and its mechanics can be altered by injuries of their components, including the patella [55] and collateral ligaments [56]. The patella acts as a fulcrum for the quadriceps tendon, enhancing the lever arm of the quadriceps muscles. In addition, patella provides stability to the knee joint by preventing lateral dislocation and improving the efficiency of quadriceps contraction [57]. However, ATT modeled in the present study is more related to ACL and meniscus than patella when knee is in extension [58]. In this study, therefore, we focused on the tibiofemoral joint with all intact tissues except for medial meniscal resection. Individuals with pre-existing knee injuries, such as ACL tears, may experience compounded effects or more significant changes in joint contact mechanics when combined with meniscectomy.

A combined 520 N axial compressive load and 104 N drawer force (or 20% × 520 N) was applied in this model study. The axial force was obtained from a measurement we used previously for the model validation when the participant applied approximately 3/4 of the body weight to his right leg [47]. While some studies suggested that vertical compression can reduce ACL laxity [59–62] and promote ACL health [63], other studies indicated that it can increase ACL tensions [15, 61, 62, 64]. The general perspective is great forces exerted on the knee joint may amplify the vulnerability to the tensile stress in ACL [15, 60]. However, the displacement of the femur (Figs. 2, 3 and 4) and the tensile stress in the ACL (Fig. 5) determined in the present study showed that medial meniscectomy could produce similar deformations in the ACL even under low-rate forces, which is supported by a published in-vitro study [60]. In their study, a combined 100 N drawer and 925 N (applied at 185 N/sec) axial compressive load was applied to the intact knee joint. The anterior-posterior translation was 5.6 mm. The present study showed a 5.4-mm anterior-posterior translation at 60 s for the total medial meniscectomized knee (Fig. 2). Another study suggested that the force of the ACL graft increases by 30–50% after medial meniscectomy under a combined 134 N drawer and 200 N axial compressive load [50]. In our case, the ACL tensile stress was increased by approximately 30% (Fig. 5) after total medial meniscectomy under a combined 108 N drawer and 520 N.

The results presented here may only be quantitively correct for the research participant we modeled. Research suggests that older patients [65] and athletes [66] may exhibit a higher prevalence of meniscectomy deformities, with gender-specific variations in degeneration and recovery rates [67]. In addition, the geometric variations within the human knee joints may play a critical role in shaping knee biomechanics [68–70]. The structural difference in the knee may be another factor influencing fluid and contact pressures. For example, contact behavior may be sensitive to meniscal transverse and cross-sectional parameters [68]. The implication of population diversity in knee joint geometry may be addressed with statistical shape modeling.

The posterior laxity was found to be more significant than the medial and distal laxities in a finite element study of an intact knee under drawer forces up to 100 N [71]. Our model, which had comparable knee alignment, force, and boundary conditions, exhibited a similar trend (Fig. 2 versus Figs. 2 and 3) as compared to that study. However, there were differences in the magnitudes of knee laxities between the results of our and their intact models. This difference may be attributed to the use of an elastic model in their study, whereas a poromechanical model in the present study.

The main limitation of this study was the absence of rotational loadings in knee joint modeling, which may produce additional strains. A comprehensive study of ACL mechanical response to knee kinematics can be performed by considering the combination of axial and rotational loadings. Another limitation was that the model predictions were not compared with the experimental data obtained from the patients with the pathological scenarios simulated. Nevertheless, the intact FE knee joint model used in this study was previously validated with in-vivo data from high-speed video-radiography measurements performed on the same participant from whom the model was built [30]. Moreover, the results of ATT and ACL strain obtained in the study were compared with similar results in the literature to show some evidence of the validity of the modeling approach (Table 2).

In conclusion, the present study investigated possible changes in ACL mechanics that may be caused by medial meniscectomy. Results suggest that increased meniscal resection may increase knee laxity and ACL stress, which have implications to collagen ruptures and altered mechanobiology in ACL under repeated daily loadings. Meniscectomy also shifted loading from the meniscus to the tibial cartilage, increasing contact pressure and shifting contact center. The findings may help understand ACL injury and its mechanobiology with a better understanding of the mechanical interactions between meniscectomy and ACL integrity. Our results may also help surgeons to choose among partial, and total meniscectomy, and meniscal repair. The modeling approach may be used to design mechanical loadings in physiotherapy for patients to best adapt the altered mechanics of their knee joints after meniscectomy.

## Data Availability

The MRI data obtained in the current study are not available for sharing due to research ethics restrictions, but model data are available from the corresponding author on reasonable request.
